# Virtual Screening as a Strategy for the Identification of Xenobiotics Disrupting Corticosteroid Action

**DOI:** 10.1371/journal.pone.0046958

**Published:** 2012-10-04

**Authors:** Lyubomir G. Nashev, Anna Vuorinen, Lukas Praxmarer, Boonrat Chantong, Diego Cereghetti, Rahel Winiger, Daniela Schuster, Alex Odermatt

**Affiliations:** 1 Swiss Center for Applied Human Toxicology and Division of Molecular and Systems Toxicology, Department of Pharmaceutical Sciences, University of Basel, Basel, Switzerland; 2 Institute of Pharmacy/Pharmaceutical Chemistry and Center for Molecular Biosciences Innsbruck – CMBI, University of Innsbruck, Innsbruck, Austria; Fudan University, China

## Abstract

**Background:**

Impaired corticosteroid action caused by genetic and environmental influence, including exposure to hazardous xenobiotics, contributes to the development and progression of metabolic diseases, cardiovascular complications and immune disorders. Novel strategies are thus needed for identifying xenobiotics that interfere with corticosteroid homeostasis. 11β-hydroxysteroid dehydrogenase 2 (11β-HSD2) and mineralocorticoid receptors (MR) are major regulators of corticosteroid action. 11β-HSD2 converts the active glucocorticoid cortisol to the inactive cortisone and protects MR from activation by glucocorticoids. 11β-HSD2 has also an essential role in the placenta to protect the fetus from high maternal cortisol concentrations.

**Methods and Principal Findings:**

We employed a previously constructed 3D-structural library of chemicals with proven and suspected endocrine disrupting effects for virtual screening using a chemical feature-based 11β-HSD pharmacophore. We tested several *in silico* predicted chemicals in a 11β-HSD2 bioassay. The identified antibiotic lasalocid and the silane-coupling agent AB110873 were found to concentration-dependently inhibit 11β-HSD2. Moreover, the silane AB110873 was shown to activate MR and stimulate mitochondrial ROS generation and the production of the proinflammatory cytokine interleukin-6 (IL-6). Finally, we constructed a MR pharmacophore, which successfully identified the silane AB110873.

**Conclusions:**

Screening of virtual chemical structure libraries can facilitate the identification of xenobiotics inhibiting 11β-HSD2 and/or activating MR. Lasalocid and AB110873 belong to new classes of 11β-HSD2 inhibitors. The silane AB110873 represents to the best of our knowledge the first industrial chemical shown to activate MR. Furthermore, the MR pharmacophore can now be used for future screening purposes.

## Introduction

Several chemicals used in agriculture and industrial production, in body care products, as food preservatives or pharmaceuticals, have been found to interfere with endocrine regulation [1], [2]. Numerous endocrine disrupting chemicals (EDCs) affecting sex steroid receptor activity have been described [3], [4], [5]. There is less known, however, on EDCs acting on corticosteroid homeostasis by disrupting the function of glucocorticoid receptors (GR), mineralocorticoid receptors (MR) or glucocorticoid metabolizing enzymes [6], [7].

Excessive MR activation, particularly when combined with high-salt diet, has been associated with renal inflammation, fibrosis, mesangial cell proliferation and podocyte injury [8]. Elevated MR activation due to enhanced local corticosteroid synthesis and impaired glucocorticoid inactivation by 11β-HSD2 have been associated with cardiovascular diseases [9], [10]. Importantly, clinical studies demonstrated a reduced morbidity and mortality in patients with acute myocardial infarction upon treatment with selective MR antagonists [10], [11]. MR is also expressed in different types of neuronal cells, and impaired MR activity has been associated with disturbed cognitive functions and behavior [12], [13].

**Figure 1:**
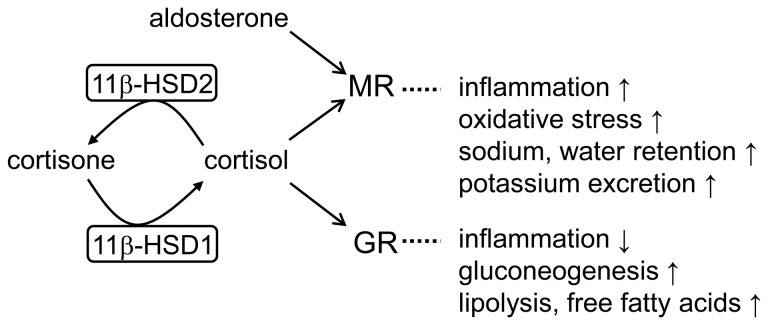
Schematic overview of corticosteroid receptor regulation by 11β-HSD enzymes.

On a cellular level, MR and GR activities are tightly regulated by 11β-HSD1 and 11β-HSD2 (Fig. 1), catalyzing the interconversion of inactive 11-ketoglucocorticoids (cortisone, 11-dehydrocorticosterone) and active 11β-hydroxyglucocorticoids (cortisol, corticosterone) [14]. Glucocorticoids and mineralocorticoids can bind with comparable affinities to MR. It is postulated that 11β-HSD2-dependent inactivation of 11β-hydroxyglucocorticoids protects MR from undesired activation by cortisol [15], [16]. Patients with loss-of-function mutations in the gene encoding 11β-HSD2 suffer from apparent mineralocorticoid excess, with hypokalemia, hypernatremia and severe hypertension [17], [18]. Inhibition of 11β-HSD2 by the licorice constituent glycyrrhetinic acid can lead to undesired cortisol-dependent MR activation [19]. Furthermore, studies with human placentas and animal studies have shown that inhibition of placental 11β-HSD2 by carbenoxolone leads to enhanced fetal glucocorticoid exposure, ultimately causing impaired metabolic and cardiovascular functions in the adulthood of the offspring [20], [21]. Recently, Deuchar et al. reported an increased progression of atherosclerosis in apolipoprotein E^−/−/^11β-HSD2^−/−^ double knock-out mice [22], whereby the MR antagonist eplerenone significantly decreased plaque formation and macrophage infiltration.

Regarding the increasing evidence for adverse health effects of 11β-HSD2 inhibition and excessive MR activation, the development of novel strategies for identifying xenobiotics that interfere with the function of these proteins is needed.

Pharmacophore-based virtual screening is a powerful strategy for predicting bioactivities of small organic molecules [23]. A pharmacophore model consists of a three-dimensional arrangement of the most important chemical functionalities for an interaction with a specific pharmacological target macromolecule [24]. It describes the locations of hydrogen bonds, hydrophobic areas, aromatic features, ionizable groups, and metal binding fragments for optimal interaction with the ligand binding site. Such a model can be applied to a large chemical database as a filter to reduce this library to only those compounds fulfilling the same interaction pattern. Virtual screening leads to an enrichment of active compounds. An initial focus on virtual hits increases the probability to find active compounds, while decreasing the number of compounds to be tested, thus saving time and costs.

This method is well established in drug discovery and has been successfully applied in lead discovery projects for various proteins [25], [26], [27], [28]. Recently, a pharmacophore-based virtual screening approach was applied for the identification of inhibitors of 17β-hydroxysteroid dehydrogenase (17β-HSD) 3 and 5 [29]. However, pharmacophore modeling and virtual screening can be also used for toxicological studies: by virtual screening *e.g.* environmental chemical databases, toxicological effects of already known chemicals can be investigated. Moreover, the toxicological potential of widely used chemicals can be studied. Recently, a pharmacophore model for 17β-hydroxysteroid dehydrogenase 3 (17β-HSD3) has been successfully employed to identify potential testosterone synthesis-disrupting compounds [30]. The virtual screening pointed towards benzophenone-type UV-filter chemicals. Systematic testing of several representatives of this class led to the identification of benzophenone-1 as a potent 17β-HSD3 inhibitor.

Here, we performed a virtual screening of a previously constructed endocrine disruptors database (EDB) [30] using a 11β-HSD pharmacophore model [31] and tested selected hits in a 11β-HSD2 bioassay. The identified silane-coupling agent AB110873 inhibited 11β-HSD2 and was further shown to activate MR. We further investigated the silane AB110873 and generated a MR pharmacophore that successfully identified AB110873.

## Results

### Virtual Screening of the EDB using the 11β-HSD Pharmacophore Model and Selection of Hits for Biological Testing

**Figure 2:**
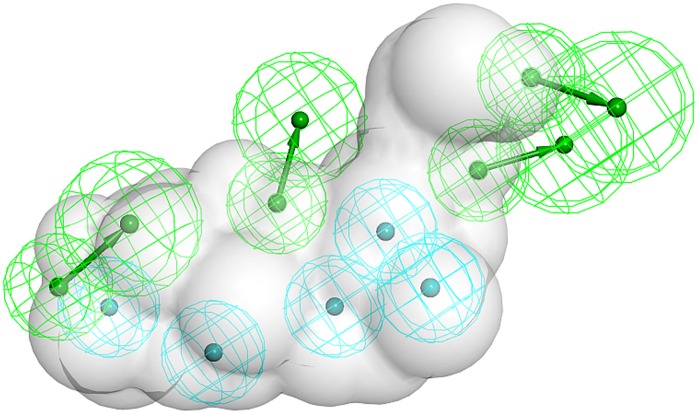
11β-HSD inhibitors pharmacophore model. The hydrogen bond acceptor features are represented in green, and the hydrophobic features in blue. The shape of carbenoxolone, as a steric constraint to prevent too large molecules from fitting, is shown as a grey volume.

In order to identify xenobiotics potentially inhibiting 11β-HSD2 we used the previously constructed endocrine disruptors database (EDB) [30] for virtual screening using an 11β-HSD pharmacophore model (Fig. 2) [31]. Out of the 76′677 database entries, only 29 fitted into the model (Table S1). The majority of the compounds represent steroids or triterpenoids, followed by antibiotics and dyes. Some of the hits are reported to be used in industry, like dyes and the silicon-containing coupling agent bis[3-(triethoxysilyl)propyl] tetrasulfide AB110873. Others are natural products with known bioactivities, especially cardiac glycosides, but also already known 11β-HSD inhibitors. For the biological testing, we had to focus on commercially available hits. From the class of cardiac glycosides, digitoxigenin monodigitoxoside was selected as representative testing hit. Because only the aglycon form was commercially available and the glycoside part of the molecule took no part in fitting to the model, digitoxigenin was actually purchased. As hit representing antibiotics lasalocid, which is used in chicken farms, was selected. Cephaeline was tested because it is a bioactive constituent of Ipecacuanha sirup, which is used as an emetic. Another natural product, hecogenin acetate, was tested because its use in cosmetics and therefore, humans are directly exposed to this agent. Also in this case, the acetate form was not available for testing, and hecogenin was biologically evaluated. Finally, AB110873 was tested as a widely used industrial chemical. Additionally, it was the only hit containing silicon; therefore it was especially interesting to evaluate this chemical composition in the context of 11β-HSD inhibition.

### Inhibition of 11β-HSD1 and 11β-HSD2 by the Selected Compounds

The selected virtual hits were tested for their potential to inhibit human 11β-HSD1 and 11β-HSD2 using lysates of stably transfected HEK-293 cells (Table 1). Glycyrrhetinic acid was used as a positive control and potently inhibited both enzymes as expected. The identification of carbenoxolone, 18α-glycyrrhetinic acid, and uralenic acid acetate, compounds shown previously to inhibit 11β-HSD1 and 11β-HSD2 [31], [32], [33], confirmed the validity of the pharmacophore used in the present study. In addition, fenofibrate, which was recently reported to inhibit mouse 11β-HSD1 activity in C2C12 cells [34], was identified by the virtual screening process and found to inhibit human 11β-HSD1 with an IC_50_ of 3.8±0.3 µM. 11β-HSD2 was not inhibited by 20 µM fenofibrate. Other compounds that were not previously reported to inhibit 11β-HSD1 include the cardiac glycoside digitoxigenin and hecogenin.

**Table 1:** Inhibition of 11β-HSD1 and 11β-HSD2 by compounds predicted by virtual screening.

Compound (20 µM)	11β-HSD1 activity (% of control)	11β-HSD2 activity (% of control)
Vehicle (0.1% dimethylsulfoxide)	100	100
T0504	N.D.	5±3
Fenofibrate	5±1	140±40
Glycyrrhetinic acid	5±3	N.D.
Digitoxigenin	36±2	104±4
Lasalocid	84±3	42±10
Hecogenin	49±9	82±2
(−) Cephaeline dihydrochloride	100±2	74±2
Bis[3-(triethoxysilyl)propyl] tetrasulfide (AB110873)	79±3	15±3

11β-HSD1-dependent conversion of cortisone (200 nM cortisone, 500 µM NADPH) to cortisol was determined by incubating lysates of HEK-293 cells stably expressing the human enzyme in the presence of vehicle or 20 µM of the respective compound for 10 min at 37°C. Similarly, 11β-HSD2-dependent conversion of cortisol (50 nM cortisol, 500 µM NAD^+^) to cortisone was measured. Data represent mean ± SD from at least three independent experiments.

T0504 and fenofibrate were used as synthetic positive controls, glycyrrhetinic acid as natural product positive control. N.D., not detectable (complete inhibition).

**Figure 3:**
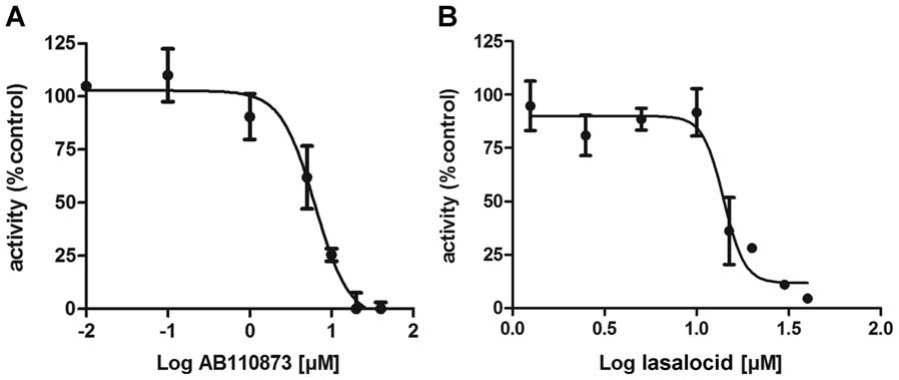
Inhibition of human 11β-HSD2 by AB110873 and lasalocid. Concentration-dependent inhibition of 11β-HSD2 by the silane-coupling agent AB110873 (*A*) and the anti-biotic lasalocid (*B*) measured in cell lysates of stably transfected HEK-293 cells. Data represent mean ± SD from three independent experiments.

Among the compounds inhibiting 11β-HSD2, the silane coupling agent AB110873 (Fig. 3) was most potent, followed by the antibiotic chemical lasalocid with IC_50_ values of 6.1±1.3 µM for AB110873 and 14±3 µM for lasalocid. Based on this first hit of a silane-coupling agent as 11β-HSD inhibitor, a related, commercially available silane was investigated. In contrast to AB110873, the silane 3-mercaptopropyl triethoxysilane, which contains only one silane group, showed very weak inhibition (80±7% remaining activity at 20 µM). In addition, we tested whether the silane AB110873 might irreversibly inhibit 11β-HSD2 and preincubated the enzyme with this compound. However, unlike inhibition of 11β-HSD2 by dithiocarbamates [35], preincubation with AB110873 did not aggravate its inhibitory effect, suggesting a reversible mode of inhibition.

### Assessment of Potential Cytotoxic Effects of the Silane AB110873

**Figure 4:**
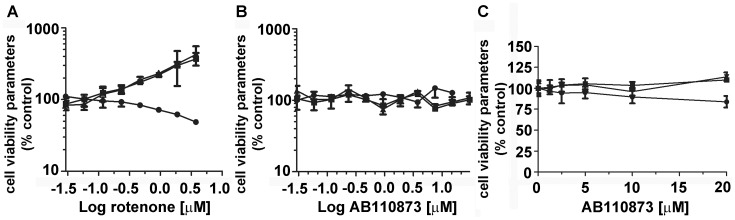
Assessment of the cytotoxic potential of the silane AB110873 and of rotenone. HEK-293 cells or BV2 cells were incubated with rotenone or AB110873 for 24 h, followed by incubation for 30 min with staining solution containing Hoechst 33342, SYTOX Green and dihydroethidium. After fixation and washing, fluorescence was analyzed using Cellomics ArrayScan high-content screening system as described in the Methods section. *A*, Effect of rotenone on cytotoxicity parameters measured in HEK-293 cells. *Filled circles*, average cell number per field; *filled squares*, cytoplasmic ROS production assessed by staining with dihydroethidium; *filled triangles*, membrane permeability assessed by SYTOX Green staining. Effect of the silane AB110873 on cytotoxicity parameters measured in HEK-293 cells (*B*) and in BV2 cells (*C*). Data show one of three independent experiments performed in eight replicates (mean ± SD, n = 8).

Next, we examined whether the silane AB110873 affects general cytotoxicity parameters. HEK-293 cells were incubated for 24 h at 37°C in the presence of vehicle or various concentrations of rotenone (Fig. 4A) or the silane AB110873 (Fig. 4B), followed by simultaneous incubation of the cells with Hoechst 33342 for staining of nuclei, DHE for detection of cytoplasmic ROS generation, and SYTOX Green to assess cell membrane permeability. Cells were analyzed using a Cellomics Array Scan high-content screening system as described in the methods section. Rotenone, a potent inhibitor of the mitochondrial electron transport chain that induces intracellular ROS production, showed a concentration-dependent decrease in the average cell number per field with a concomitant increase in plasma membrane permeability and the production of cytoplasmic ROS (Fig. 4A). The silane AB110873 at concentrations up to 30 µM had no significant effect (Fig. 4B). Similarly, the silane AB110873 did not affect these general cytotoxicity parameters in monkey kidney COS-1 cells (data not shown) and in BV2 microglial cells (Fig. 4C), which express endogenous MR.

### Activation of MR by the Silane AB110873

**Figure 5:**
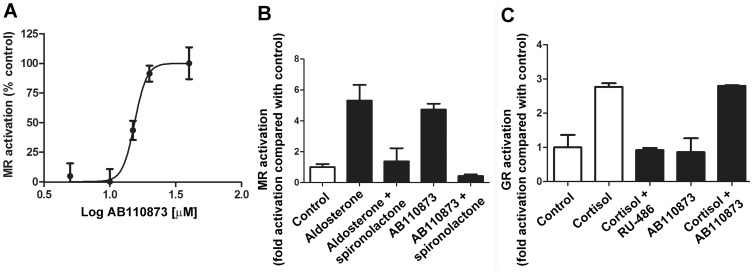
Effect of the silane AB110873 on the activation of mineralocorticoid (MR) and glucocorticoid receptors (GR). The impact of silane AB110873 on MR and GR transactivation was measured in HEK-293 cells transfected with MMTV-LacZ reporter, MR (*A,B*) or GR (*C*) and luciferase transfection control. Cells were incubated with the receptor ligands for 24 h, followed by analysis of reporter activity as given in the Methods section. *A*, Concentration-dependent activation of the human MR by the silane AB110873. Maximal activation at 30 µM was set as 100%. *B*, effect of the antagonist spironolactone (1 µM) on MR activation by aldosterone (5 nM) or by the silane AB110873 (20 µM); *C*, activation of human GR by cortisol (100 nM) and effect of AB110873 (20 µM) and antagonist RU-486 (1 µM) (MR and GR activity of the vehicle control was set as 1, data represent fold activation). Data were obtained from four independent experiments, each performed in triplicates (mean ± SD).

To investigate whether the silane AB110873 directly modulates MR activity, we performed transactivation assays using HEK-293 cells transfected with human MR and a galactosidase reporter gene under the control of the MR sensitive MMTV-promoter. The silane AB110873 stimulated reporter gene activity in a concentration-dependent manner (Fig. 5A) with an EC_50_ of 15.4±0.5 µM. Maximal reporter gene stimulation obtained with AB110873 was comparable to maximal activation observed with aldosterone (Fig. 5B). Importantly, the MR antagonist spironolactone was able to completely abolish receptor activation by AB110873 (Fig 5B). In contrast, the silane AB110873 did not activate the human GR and was unable to block GR activation mediated by cortisol (Fig 5C). The 3-mercaptopropyl triethoxysilane did not activate MR at concentrations up to 30 µM (data not shown).

### The Silane AB110873 Increases Mitochondrial Superoxide Generation and Induces IL-6 Expression through MR Activation in BV-2 Microglial Cells

**Figure 6:**
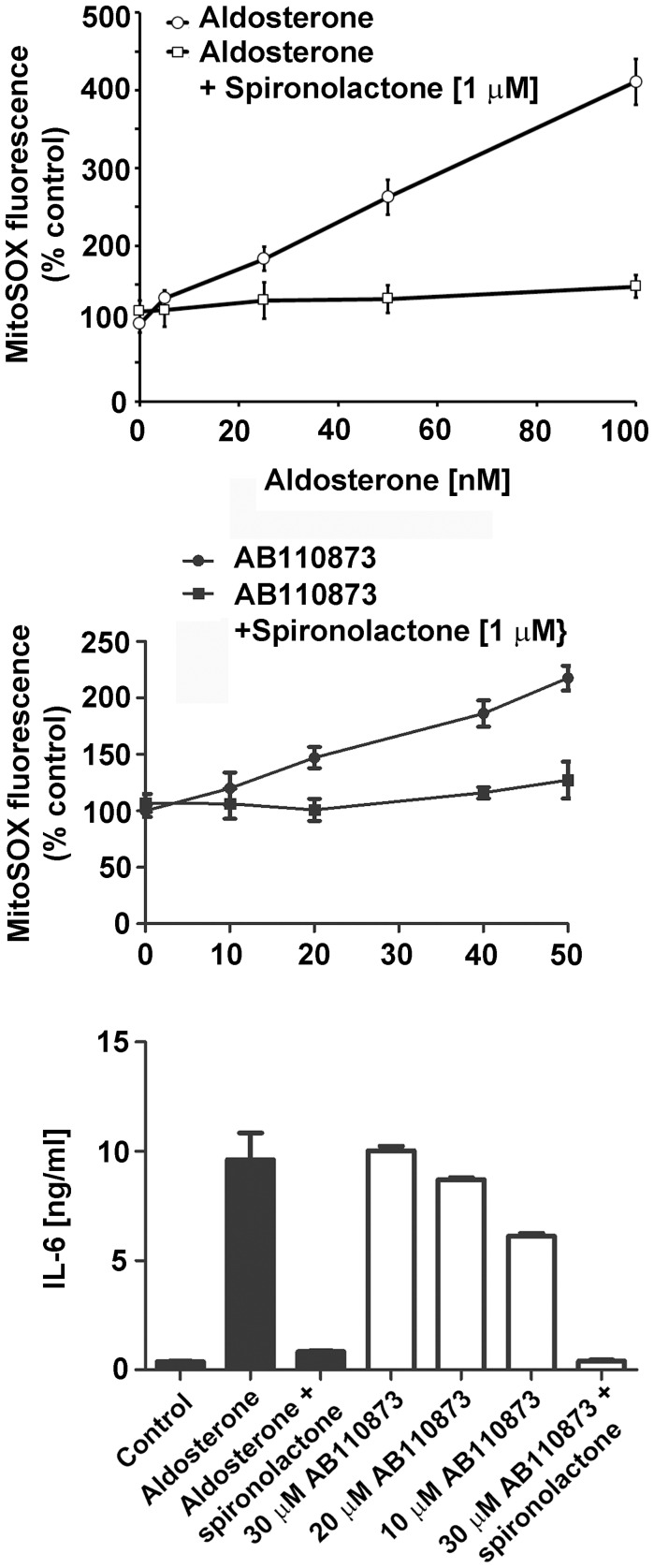
Increased mitochondrial superoxide generation and induction of IL-6 production upon MR activation in BV2 cells. BV2 microglial cells were incubated for 24 h with increasing concentrations of aldosterone (*A*) or silane AB110873 (*B*) in the presence or absence of 1 µM spironolactone, followed by determination of mitochondrial superoxide using MitoSox staining as described in the Methods section. Fluorescence was analyzed using a Cellomics ArrayScan high-content screening system. *C*, BV2 cells were incubated for 24 h with aldosterone (5 nM) or various concentrations of silane AB110873 in the presence or absence of spironolactone, followed by quantification of IL-6 protein levels in the medium of the cultured cells by ELISA. Data (mean ± SD) were obtained from three independent experiments each performed in six replicates.

MR activation in macrophage-derived cells has been associated with an elevation in mitochondrial ROS production. Therefore, we investigated whether aldosterone and the silane AB110873 might activate MR in mouse microglial BV2 cells that express endogenous levels of the receptor. Incubation of BV2 cells for 24 h with aldosterone, followed by MitoSox staining, revealed increased mitochondrial superoxide formation, an effect which was abolished by coincubation with the MR antagonist spironolactone (Fig. 6A). This confirms the functional expression of MR in microglial BV2 cells. Similarly, the silane AB110873 enhanced mitochondrial superoxide generation by a concentration-dependent manner in BV2 cells, and spironolactone was able to almost fully prevent mitochondrial ROS production (Fig. 6B).

Previous reports associated MR activation with an increased expression of the pro-inflammatory cytokine IL-6 [36], [37]. In microglial BV-2 cells aldosterone at 50 nM led to increased IL-6 protein levels, which was suppressed by spironolactone (Fig 6C). The silane AB110873 stimulated IL-6 protein expression by a concentration-dependent manner. At 30 µM AB110873 IL-6 levels were comparable with those of aldosterone treated cells. Importantly, spironolactone almost completely reversed the effect of AB110873. The 3-mercaptopropyl triethoxysilane did not affect mitochondrial superoxide generation and IL-6 protein expression (data not shown).

### Generation of an MR Ligands Pharmacophore Model

**Figure 7:**
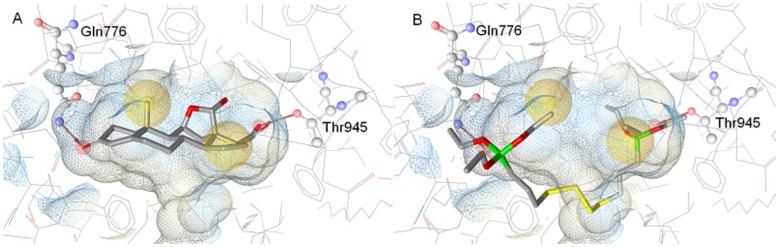
Binding of aldosterone and the silane AB110873 to the MR. *A*, MR pharmacophore model with the cocrystallized ligand aldosterone. *B,* the silane AB110873 fitted to the MR ligand binding site with the pharmacophore model. Hydrogen bond acceptors are shown as red arrows, hydrophobic areas as yellow spheres. Exclusion volumes are not shown for clarity. Receptor binding pocket is colored by aggregated lipophilicity (grey)/hydrophilicity (blue).

A structure-based pharmacophore model for MR ligands was generated based on the physicochemical interactions between the endogenous MR agonist aldosterone and the MR receptor. For this purpose, interaction information was derived from an X-ray crystal structure of the two binding partners (PDB entry 2aa2). All observed interactions were translated into an interaction model consisting of seven features: four hydrogen bond acceptors with Asn770, Cys942, Gln776, and Thr945, from which the latter is defined as a bidirectional hydrogen bond specified as an additional hydrogen bond donor feature with this residue, and two hydrophobic features placed on the methyl group of aldosterone and to the ring D of steroidal core. The model was used to virtually screen a set of active and inactive MR ligands from several publicly accessible databases (see experimental section). Unfortunately, this model was too restrictive and not able to correctly identify a large fraction of active ligands throughout all datasets. In order to better represent the chemical features essential for MR binding, four of the features were removed. The final MR ligands model consisted of four features: two hydrogen bond acceptors (with Gln776 and Thr945) and two hydrophobic features (Fig. 7A). Exclusion volumes - forbidden areas for the ligand due to steric clashes with the protein - were added to represent the shape of the binding site. This new model showed an improved recognition rate of MR ligands and was therefore used for further experiments. To explore how the silane AB110873 would bind to the MR, it was fitted first into the MR pharmacophore model. The silane fits to the MR model with a high fit score of 46.13, the maximum possible fit score being 49.00. Unfortunately, when aligning the silane AB110873 with the pharmacophore into the MR ligand binding pocket, some severe steric clashes were observed. Additionally, AB110873 sticked out from the binding pocket (Fig. 7B). This suggested that silane AB110873 may adopt a different binding orientation within the MR binding site compared to aldosterone.

### Docking Studies on AB110873 Fitting into the 11β-HSD2 and MR Binding Sites

**Figure 8:**
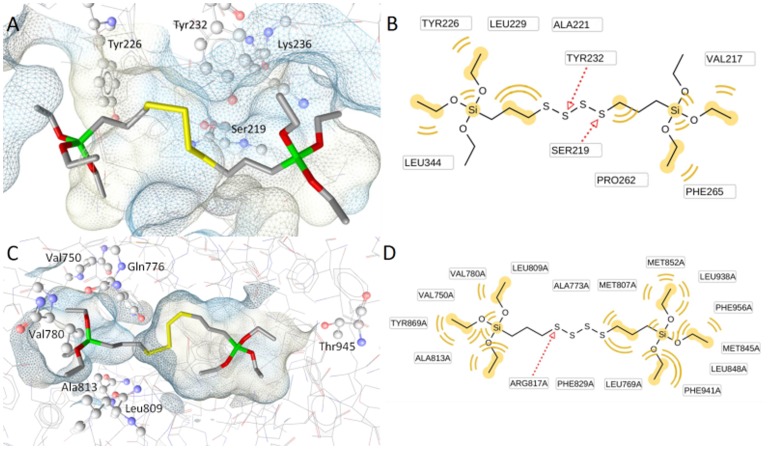
AB110873 binds to both 11β-HSD2 and MR. Proposed binding modes of AB110873 in 11β-HSD2 (*A,B*) and in the MR (*C,D*). In the 3D-figures, selected amino acid residues are highlighted with ball and stick, and the receptor binding pockets are colored by aggregated lipophilicity (grey)/hydrophilicity (blue). The 2D figures represent the binding interactions, color-coded as following: red arrow – hydrogen bond acceptor, yellow – hydrophobic interaction.

The silane AB110873 is anchored mostly via hydrophobic interactions to the ligand binding site of 11β-HSD2 (Fig. 8A,B). Two hydrogen bonds are formed between the sulfur bridge and the catalytic residues Tyr232 and Ser219. Because of the chemical nature of sulfur, these hydrogen bonds are not especially strong, and there are no other stronger interactions to fix the silane AB110873 in the vicinity of the catalytic residues. Therefore, the inhibitory activity could be explained by two ways: first, the hydrogen bonds disrupt the catalytic function and/or, second, the bulky silane AB110873 prevents any other ligand binding via steric effects.

The docking studies of silane AB110873 in MR suggested an interesting binding mode that extends the one of aldosterone. Silane AB110873 binds to the ligand binding pocket with similar hydrophobic interactions compared to aldosterone, but it additionally occupies a hydrophobic side pocket formed by Val750, Val780, Leu809, and Ala813 (Fig. 8C). Remarkably, the channel between these two pockets is constricted and the hydrophobic side pocket is buried in the receptor.

**Figure 9:**
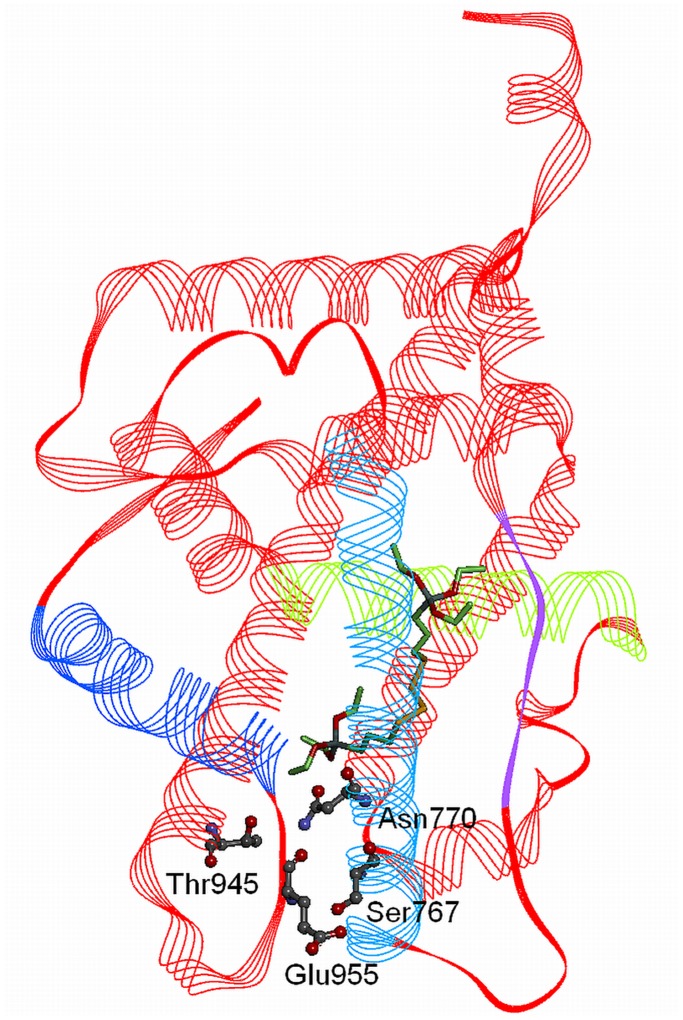
MR with silane AB110873. The amino acids that form the hydrogen bond network with steroidal agonists are highlighted in ball and stick style. AF-2 (helix 12) is colored in dark blue, helix 5 in green, helix 3 in light blue, and the β-sheet in lilac. The buried hydrophobic pocket additionally occupied by AB110873 is formed by amino acids from helices 3 (Val780) and 5 (Leu809, Ala813), and from the β-sheet (Val750).

The automatically calculated ligand-protein interactions by LigandScout revealed that silane AB110873 binds to the MR by hydrophobic interactions (Fig. 8D). The only hydrogen bond is formed between Arg817 and a sulfur atom. The silane AB110873 has some common hydrophobic interactions with Phe941, Leu938, Met845, Met807, and Ala773 as the original agonist aldosterone. However, it does not form the typical hydrogen bonding network of steroidal agonists described by Bledsoe et al. (Fig. 9) [38]. In addition, the hydrophobic side pocket that the silane AB110873 is occupying is located between helixes 3 and 5 as well as a β-sheet (Fig. 9). Therefore, the silane AB110873 is not interfering with the MR-coactivator peptide.

## Discussion

In the present study, we describe a novel approach using *in silico* and *in vitro* tools for the rapid screening of large numbers of compounds in order to identify and characterize xenobiotics that inhibit 11β-HSD2 or act directly on MR. In this proof-of-concept study, the previously constructed endocrine disruptor database (EDB), containing approximately 80,000 compounds [30], was screened against an 11β-HSD pharmacophore. Of five compounds predicted to bind to 11β-HSDs and that were chosen for biological testing, four showed more than 50% inhibition of one of the two 11β-HSD enzymes. Thus, the *in silico* screening had a very high hit rate, compared with high-content screening, and led to the identification of non-steroidal and non-triterpenoid compounds that have not been reported before. The *in silico* tools used in this study have thereby shown high predictive power and can be subsequently used in future screening projects, *e.g.* using virtual natural compound libraries to identify compounds with glycyrrhetinic acid-like effects or virtual drug libraries to detect unwanted side-effects.

Due to its low general cytotoxicity, also when compared with other hits of the *in silico* screening, and its rather unexpected structure compared with known 11β-HSD2 inhibitors, the silane coupling agent AB110873 was further investigated. Silane derivatives are used at concentrations higher than 1% in resin-composite cement [39]. They are also used in fluoride varnishes for caries prevention [40], and silane-based hydrogels are used in clinical practice for delivery of nitric oxide donors [41]. The specific silane coupling agent AB110873, also known as KH-69 or Si-69, is one of the most widely used rubber additives, developed thirty years ago (http://www.rubber-silanes.com/product/rubber-silanes/en/about/pages/default.aspx). AB110873 leads to improved strength and optimized dynamic properties of rubbers, including increased abrasion resistance. It is used in many applications where white fillers containing silanol groups are involved and optimum technical properties are required. AB110873 is widely used in the production of tires, shoe soles, mechanical rubber goods, and as adhesion promoter for rubber adhesives, sealants and coatings. Further, AB110873 and other bis-type silanes are used to protect metals from corrosion [42]. Despite of the wide use of AB110873, there are currently no studies available on concentrations measured in humans. Regarding the expected low release of silane derivatives from their products, occupational exposure during the production process probably represents the most critical issue.

Nevertheless, our results show that the silane AB110873 at subcytotoxic concentrations can enhance MR activity by two distinct mechanisms: first, by inhibition of 11β-HSD2, thereby leading to locally increased levels of active glucocorticoids that activate MR, and second, by direct activation of the receptor. Inhibition of 11β-HSD2 is relevant in classical mineralocorticoid-responsive cells involved in water and electrolyte regulation that coexpress MR and 11β-HSD2 such as renal cortical collecting ducts, distal colon and salivary and sweat glands [14]. It is further relevant in the placenta as a barrier to protect the fetus from maternal glucocorticoids.

A direct activation of MR is further relevant in cells not expressing 11β-HSD2. MR and GR are coexpressed in the presence of the glucocorticoid activating enzyme 11β-HSD1 in cells like macrophage and microglia, adipocytes and osteoblasts [14], where an impaired MR activation has been associated with elevated inflammation and oxidative stress [43], [44]. Thus, a chronic exposure to MR activating chemicals can be expected to result in exaggerated inflammatory response and impaired ability to cope with oxidative stress. Our results show that the silane AB110873 selectively binds to MR but not GR, and molecular modeling revealed a binding mode distinct to that of aldosterone. The finding that AB110873 occupies an additional hypdrophobic side pocket of MR may offer an opportunity for drug development, since MR antagonists are of considerable pharmaceutical interest.

In conclusion, virtual screening of the EDB using a 11β-HSD pharmacophore led to the identification of non-steroidal and non-triterpenoid xenobiotics, including the antibiotic lasalocid and the silane AB110873. The latter was further shown to activate recombinant and endogenous MR. Molecular modeling revealed the binding mode of AB110873 in the ligand binding pocket, explaining the fact that AB110873-mediated MR activation was blocked by spironolactone. Finally, a newly generated MR pharmacophore successfully recognized AB110873, indicating its value for future screening experiment.

## Materials and Methods

### Materials

[1,2,6,7-^3^H]-cortisol was purchased from PerkinElmer (Waltham, MA), [1,2,6,7-^3^H]-cortisone from American Radiolabeled Chemicals (St.Louis, MO), 5H-1,2,4-triazolo(4,3a)azepine,6,7,8,9-tetrahydro-3-tricyclo(3·3·1·13·7)dec-1-yl (T0504) from Enamine (Kiev, Ukraine) and aldosterone from Steraloids (Wilton, NH). Digitoxigenin, lasalocid, and (-)cephaeline dihydrochloride were kindly provided by the National Cancer Institute (NCI, Bethesda, US). All other chemicals were from Fluka AG (Buchs, Switzerland) of the highest grade available. Cell culture media were purchased from Sigma Aldrich (Buchs, Switzerland).

### Inhibitor-based 11β-HSD2 Pharmacophore Model and Virtual Screening of a 3D-structure Database of Potential Endocrine Disrupting Chemicals (EDCs)

For this study, a previously reported and experimentally validated 11β-HSD inhibitor pharmacophore model was used [31]. The model was based on two triterpenoid 11β-HSD inhibitors with preference to inhibit 11β-HSD2 more potently than 11β-HSD1. The model consisted of five hydrophobic features, placed on the triterpene core of the training compounds, and four hydrogen bond acceptors (Fig. 2).

The EDB was screened with the 11β-HSD2 inhibitors model using Catalyst 4.11 (www.accelrys.com). For the virtual screening, the best flexible search algorithm was used. This screening protocol minimizes the pre-computed compound conformations from the screening database into the model in order to find the best fittings also for flexible compounds.

### Cell Culture

HEK-293 (CRL-1573) and COS-1 (CRL-1650) cells were purchased from American Type Culture Collection (Manannas, VA, USA) and cultured in Dulbecco's modified Eagle medium (DMEM) supplemented with 10% fetal bovine serum (FBS), 4.5 g/L glucose, 50 U/mL penicillin/streptomycin, 2 mM glutamine, and 1 mM HEPES, pH 7.4. They were used at passage number between 5 and 30. Immortalized mouse microglia BV2 cells, developed by Bocchini et al. [45], were a kind gift from Dr. Ernst Malle (University of Graz, Graz, Austria) and were cultivated in RPMI-1640 medium, supplemented with 10% fetal bovine serum, 1 g/L glucose, 100 U/mL penicillin, 100 µg/mL streptomycin, 2 mM glutamine and 0.1 mM non-essential amino acids. BV2 cells were used at passage number between 7 and 15.

### 11β-HSD1 and 11β-HSD2 Activity Assay

Enzyme activities were measured as described earlier using lysates of HEK-293 cells stably expressing human 11β-HSD1 or 11β-HSD2 [46]. 11β-HSD1 oxoreductase activity was determined by incubation of lysates with 200 nM of radiolabeled cortisone, 500 µM NADPH and vehicle or inhibitor. 11β-HSD2 activity was measured in the presence of 50 nM radiolabeled cortisol, 500 µM NAD^+^ and vehicle (dimethylsulfoxide (DMSO)) or inhibitor. Samples were incubated for 10 min at 37°C. Control samples in the presence of vehicle converted 20% of the radiolabeled substrate. Data represent percentage of activity of the enzyme in the presence of test compound relative to its activity (set as 100%) in the presence of vehicle control.

### MR and GR Transactivation Assay

HEK-293 cells (150,000 cells/well) were seeded in poly-L-lysine coated 24-well plates and incubated for 24 h. Cells were transfected using calcium phosphate precipitation with pMMTV-lacZ β-galactosidase reporter (0.20 µg/well), pCMV-LUC luciferase transfection control (0.04 µg/well) and plasmid for human recombinant MR or GR (0.35 µg/well). The medium was changed 6 h post-transfection, followed by incubation for another 18 h. Cells were washed twice with charcoal-treated DMEM containing 10% FBS and incubated with either 10 nM aldosterone or 100 nM cortisol and various concentrations of compounds for 24 h to allow sufficient reporter gene expression. The solvent concentration was below 0.1%. Cells were washed once with PBS and lysed with 50 µL lysis buffer of the Tropix kit (Applied Biosystems, Foster City, CA) supplemented with 0.5 mM dithiothreitol. Lysed samples were frozen at −80°C prior to analysis of β-galactosidase activity using the Tropix kit and luciferase activity using a luciferine-solution [46]. Relative light units (RLU) of 4,100 and 195,000 for luciferase and β-galactosidase activity were obtained for vehicle controls. The RLU of luciferase control was not significantly altered by treatment with compounds. Data were normalized to the ratio of β-galactosidase to luciferase activity of the vehicle control.

### Determination of Cellular Toxicity Parameters and Cytoplasmic ROS Production

HEK-293 and COS-1 cells (9′000 cells/well) were seeded in poly-L-lysine coated 96-well plates and allow to attach for 24 h prior to incubation with compounds for another 24 h. Upon addition of 50 µL staining solution (DMEM containing 2.5 µM SytoxGreen, 500 nM Hoechst 33342 and 2.5 µg/mL dihydroethidium (DHE)) cells were incubated for 30 min at 37°C. The solution was aspirated and cells were fixed with 3.5% paraformaldeyde in PBS for 15 min. Cells were washed three times with PBS and plates stored at 4°C until analysis using a Cellomics ArrayScan high-content screening system (Cellomics ThermoScientific, Pittsburgh, PA). Data acquisition and image analysis was performed by automated fluorescence imaging. Fluorescence intensity measurements (population averages) were captured on 20 fields, each field containing approximately 1000 cells. Values were normalized to vehicle control and data represent percentage of fluorescence intensity compared with control. Images were acquired for each fluorescence channel (Hoechst 33342, SytoxGreen, DHE) using suitable filters, a 20× objective and the application software according to the manufacturer. Cells were identified using Hoechst 33342, and a nuclear mask was generated from images of stained nuclei. Automatic focusing was performed in the nuclear channel to ensure focusing regardless of staining intensities in the other channels. Images and data regarding intensity and texture of the fluorescence within each cell as well as average fluorescence of the cell population within the well were quantified from a shell surrounding the nuclear mask.

The MTT assay was performed with 23′000 cells/well seeded in poly-L-lysine coated 96-well plates. After overnight incubation, the medium was replaced and cells were incubated with compounds for 24 h, followed by adding 5 mg/mL MTT and examination for appearance of purple formazan crystals using light microscopy after another 2 h. The medium was carefully aspirated and 200 µL DMSO were added to each well to dissolve the formazan crystals. After 30 min, the plate was measured at 565 nm with a reference filter at 650 nm.

### Measurement of Mitochondrial Superoxide Production in BV2 Cells

To determine whether compounds affected steady-state levels of mitochondrial superoxide production in BV2 microglial cells that express endogenous MR, the cationic superoxide-sensitive dye MitoSOX Red was used. Cells were washed once with PBS and labeled with MitoSOX Red (5 µM) at 37°C for 20 min. Cells were washed with PBS and fixed with 3.5% paraformaldehyde for 15 min, followed by washing and incubation with nuclei staining solution (1 µg/ml Hoechst 33342 dissolved in medium) for 30 min. The plate was washed twice with PBS, cells were imaged and the amount of fluorescence was quantified using an ArrayScan high-content screening system. Fluorescence intensity measurements (population averages) at 590 nm were captured on 20 fields with each field containing approximately 1000 cells. The amount of mitochondrial superoxide production is given as percentage of fluorescence intensity compared with control.

### Detection of Interleukin-6 (IL-6) Expression by Enzyme-linked Immunosorbent Assay (ELISA)

IL-6 protein in culture media from BV2 microglial cells was measured using the IL-6 ELISA Ready-SET-Go kit (BD Biosciences, CA) according to the manufacturer. Cells were seeded in 96-well plates at a density of 5×10^4^ cell/mL. Cell-free supernatants were collected after incubation for 24 h with AB110873 and aldosterone in the presence or absence of spironolactone. The 96-well plate was incubated overnight at 4°C with coating buffer containing capture antibody. Samples were washed 5 times with PBS-T (PBS containing 0.05% Tween-20), blocked for 1 h, washed again, and standards and collected culture media were added to the appropriate wells and incubated for 2 h. After washing 5 times with PBS-T, each well was incubated with detection antibody for 1 h, washed and incubated with avidin–horseradish peroxidased for 30 min. After washing 7 times, samples were incubated with substrate solution for 30 min in the dark. Reactions were stopped with 1 M H_3_PO_4_, and the plate was analyzed at 450 nm by subtraction of the values obtained at 570 nm using a UV-max kinetic microplate reader (Molecular Device, Birkshire, UK). The concentrations of IL-6 were calculated according to the standard curve of purified mouse IL-6.

### Generation of a MR Ligands Pharmacophore Model

The structure-based MR ligands pharmacophore model was generated using LigandScout 2.02 ( [47]; www.inteligand.com). The model was based on the interactions of the MR binding site and its cocrystallized endogenous ligand aldosterone (Protein Data Bank (PDB) code 2aa2, [38]). The pharmacophore model was validated by virtual screening of MR data sets from the directory of useful decoys (DUD) [48], the ChEMBL database version 02 (www.ebi.ac.uk/chembl/), and a previously reported drug-like virtual library [49]. To analyze whether the silane AB110873 fits to the MR pharmacophore model, a database of 500 conformations of this molecule was calculated with Omega-best settings using the idbgen-tool of LigandScout, followed by screening the MR model against this database using LigandScout 3.03a [47].

### Docking Studies

To propose the molecular binding modes of the most active compound to the 11β-HSD2 and MR binding sites, docking studies using GOLD software [50], [51] were performed. To generate the binding modes, GOLD uses a genetic algorithm, which creates ten low-energy binding solutions for each ligand within the binding site. No further energy minimization for the predicted binding modes was performed. In the absence of an X-ray crystal structure of 11β-HSD2, a previously published homology model of 11β-HSD2 [46] was used. The binding site was defined as a 15 Å sphere with the Tyr232 oxygen as center. GoldSscore was used as a scoring function and ten docking poses were reported for the ligand. The program was set to define the atom types of the protein and the ligand automatically. For the MR, the PDB entry 2aa2 [38] was selected. The binding site was again defined as a 15 Å sphere, which center was the oxygen atom of Asn770. The same settings were applied as in the 11β-HSD2 docking run. Using these settings, the program successfully reproduced the binding mode of the cocrystallized ligand aldosterone. After both docking experiments, LigandScout [47] was used for analyzing the predicted protein-ligand interactions.

## Supporting Information

Table S1 Virtual hits derived from the EDB by virtual screening of the 11β-HSD pharmacophore.
(DOC)
