# Supplementary material for: Virtual Screening as a Strategy for the Identification of Xenobiotics Disrupting Corticosteroid Action
Source: PLoS One. 2012 Oct 4;7(10):e46958. doi: 10.1371/journal.pone.0046958 (PMC3464284; doi:10.1371/journal.pone.0046958)
Supplement: Table S1 — Virtual hits derived from the EDB by virtual screening of the 11β-HSD pharmacophore. (DOC) [file pone.0046958.s001.doc]

**Table S1**

Virtual hits derived from the EDB by virtual screening with the 11β-HSD2 pharmacophore model

|  |  |
| --- | --- |
| Ethanesulfonic acid, CAS17011-51-7,  patented fungicide | Carbenoxolone, CAS5697-56-3,  known unselective 11β-HSD inhibitor |
|  |  |
| CAS783252-38-0, no references | Peruvoside, CAS1182-87-2,  cardiac glycoside |
|  |  |
| 18α-glycyrrhetinic acid, CAS 1449-05-4,  already known 11β-HSD inhibitor | Jervine acetate, CAS 14788-78-4,  alkaloid in *Veratrum album*, antihypertensive |
|  |  |
| Dexamethasone m-sulfobenzoate, CAS16978-57-7, used for preparation of parenteral medicaments | Digitoxigenin monodigitoxoside, glycoside  CAS18404-43-8, cardiac glycoside, potential antitumor use |
|  |  |
| Ardisiachinon A, CAS 18799-05-8,  5-LOX inhibitor, antiallergic effects | CAS23394-07-2, cardiac glycoside |
|  |  |
| CAS23370-68-5, reactant in a fabric softener synthesis | Lasalocid, CAS 25999-31-9, antibiotic, accumulates to chicken eggs |
|  |  |
| CAS28535-81-1, reactant in synthesis of steroidal HIV-1 protease inhibitors | Benzopurpurin 10B, CAS 25188-28-7,  Congo Red analogue |
|  |  |
| Beaumontoside, CAS 31087-87-3,  cardiac glycoside | Silane AB110873, CAS 40372-72-3, used as a rubber additive (http://www.rubber-silanes.com/product/rubber-silanes/en/about/pages/default.aspx) |
|  |  |
| Hecogenin acetate, CAS 915-35-5,  cosmetic use | Oleandrin, CAS 465-16-7, cardiac glycoside, potential neuroprotective agent |
|  |  |
| Veratrosine, CAS 475-00-3, alkaloid from *Veratrum dahuricum*, showing weak antitumor activity | Evomonoside, CAS 508-93-0,  cardiac glycoside |
|  |  |
| CAS 53735-71-0,  cardiac glycoside | CAS 53735-73-2,  cardiac glycoside |
|  |  |
| Cephaeline (HCl), CAS 483-17-0, component on the extract of Ipecacuanha, Ipecac syrup has emetic use | Uralenic acid acetate, CAS 6277-14-1,  known 11β-HSD inhibitor |
|  |  |
| Ursodeoxycholylglycine, CAS 64480-66-6  bile acid conjugate | Benzenesulfoic acid, CAS 66182-97-6,  used in photography |
|  |  |
| CAS 68527-98-0, reactant in chemical synthesis | CAS70146-06-4  No references |
|  |  |
| CAS83027-46-7, red color (Acid red 118) |  |

**References**

Besne, I. (2003). Use of sapogenin or sapogenin-containing plant extract for smoothing skin lines and wrinkles. *EP patent 1352643 A1 2003101*.

Classen-Houben, D., Schuster, D., Da Cunha, T., Odermatt, A., Wolber, G., Jordis, U. and Kueenburg, B. (2009). Selective inhibition of 11beta-hydroxysteroid dehydrogenase 1 by 18alpha-glycyrrhetinic acid but not 18beta-glycyrrhetinic acid. *J Steroid Biochem Mol Biol* **113**, 248-52.

Deigner, H.-P., Kohl, M., Koal, T., Igwe, I. E., Dallmann, G., Bauer, M. and Enot, D. (2011). Use of bile acids for prediction of an onset of sepsis. *WO patent 2011157655 A1 20111222*.

Diederich, S., Grossmann, C., Hanke, B., Quinkler, M., Herrmann, M., Bahr, V. and Oelkers, W. (2000). In the search for specific inhibitors of human 11beta-hydroxysteroid-dehydrogenases (11beta-HSDs): chenodeoxycholic acid selectively inhibits 11beta-HSD-I. *Eur J Endocrinol* **142**, 200-7.

Dunn, D. E., He, D. N., Yang, P. Y., Johansen, M., Newman, R. A. and Lo, D. C. (2011). In vitro and in vivo neuroprotective activity of the cardiac glycoside oleandrin from Nerium oleander in brain slice-based stroke models. *J Neurochem* **119**, 805-14.

Fukuishi, N., Takada, T., Fukuyama, Y. and Akagi, M. (2001). Antiallergic effect of ardisiaquinone A, a potent 5-lipoxygenase inhibitor. *Phytomedicine* **8**, 460-64.

Gilani, A. H., Aftab, K., Saeed, S. A., Ali, R. A. and Rahman, A. U. (1995). O-Acetyljervine - a New Beta-Adrenoceptor Agonist from Veratrum-Album. *Arch Pharm Res* **18**, 129-32.

Gomes, A. C., Nunes, J. C. and Simoes, R. M. (2010). Determination of fast ozone oxidation rate for textile dyes by using a continuous quench-flow system. *J Hazard Mater* **178**, 57-65.

Hashimoto, T., Rathore, H., Satoh, D., Hong, G., Griffin, J. F., From, A. H. L., Ahmed, K. and Fullerton, D. S. (1986). Cardiac-Glycosides .6. Gitoxigenin C16-Acetates, Formates, Methoxycarbonates, and Digitoxosides - Synthesis and Na+,K+-Atpase Inhibitory Activities. *J Med Chem* **29**, 997-1003.

Jelinek, C. F., Stanley, L. N. and Armento, W. H. (1964). Diazoamino fungicides. *US patent 3138521 19640623*.

Jensen, M., Schmidt, S., Fedosova, N. U., Mollenhauer, J. and Jensen, H. H. (2011). Synthesis and evaluation of cardiac glycoside mimics as potential anticancer drugs. *Bioorg Med Chem* **19**, 2407-17.

Krasso, A. F., Weiss, E. K. and Reichstein, T. (1963). Cardenolides from beaumontia gradiflora. 2. Elucidation of structure of wallichoside, beaumontoside and beauwalloside. *Helvetia Chimica Acta* **46**, 1693-96.

Moeller, A., Roos, C., Wagner, R., Sockel, K.-H., Stachulla, K.-H., Witosse, A. and Lange, H. (2006). *WO patent 2006103075 A1 20061005*.

Moormann, J. and Rotermund, C. (1993). *EP patent 545212 A1 19930609*.

Nanba, T., Uemura, T. and Fujikawa, T. (2003). JP patent 2003012678 A 20030115.

Rudyk, H., Knaggs, M. H., Vasiljevic, S., Hope, J., Birkett, C. and Gilbert, I. H. (2003). Synthesis and evaluation of analogues of congo red as potential compounds against transmissible spongiform. encephalopathies. *Eur J Med Chem* **38**, 567-79.

Salunke, D. B., Hazra, B. G., Gonnade, R. G., Bhadbhade, M. M. and Pore, V. S. (2005). An efficient method for the synthesis of methyl 11 alpha-amino-3 alpha,7 alpha-diacetoxy-12-oxo-5 beta-cholan-24-oate. *Tetrahedron* **61**, 3605-12.

Schuster, D., Maurer, E. M., Laggner, C., Nashev, L. G., Wilckens, T., Langer, T. and Odermatt, A. (2006). The discovery of new 11beta-hydroxysteroid dehydrogenase type 1 inhibitors by common feature pharmacophore modeling and virtual screening. *J Med Chem* **49**, 3454-66.

Sturm, S., Taglioli, V., Bilia, A. R., Vincieri, F. and Stuppner, H. (2003). Analysis of alkaloids in Ipecacuanhae radix and preparations by capillary zone electrophoresis. *J Sep Sci* **26**, 1175-79.

Takechi, M. and Tanaka, Y. (1994). Structure-Activity-Relationships of Synthetic Digitoxigenyl Glycosides. *Phytochemistry* **37**, 1421-23.

Tang, J., Li, H. L., Shen, Y. H., Jin, H. Z., Yan, S. K., Liu, R. H. and Zhang, W. D. (2008). Antitumor activity of extracts and compounds from the rhizomes of Veratrum dahuricum. *Phytother Res* **22**, 1093-96.

Visconte, G. W., Bagchi, P., Friday, J. A., Orem, M. W. and Pitt, A. R. (1994). Viscosity control of photographic melts. *US patent 5300148 A 19940405*.

Wang, L., Wible, B. A., Wan, X. P. and Ficker, E. (2007). Cardiac glycosides as novel inhibitors of human ether-a-go-go-related gene channel trafficking. *J Pharmacol Exp Therap* **320**, 525-34.

Wong, R. Y. P. and Roxburgh, J. W. (2010). Lasalocid awareness and sampling in Scotland. *Int J Environ Heal R* **20**, 159-69.
